# Association between social trust and the risk of cardiovascular disease in older adults in Korea: a nationwide retrospective cohort study

**DOI:** 10.1186/s12889-020-09964-z

**Published:** 2020-12-01

**Authors:** Seo Eun Hwang, Seulggie Choi, Kyuwoong Kim, Jong-Koo Lee, Juhwan Oh, Sang Min Park

**Affiliations:** 1 Department of Family Medicine, Seoul National University- Seoul Metropolitan Government Boramae Medical Center, Seoul, South Korea; 2 Department of Medicine, Seoul National University Graduate School, Seoul, South Korea; 3 Department of Biomedical Sciences, Seoul National University Graduate School, Seoul, South Korea; 4 Department of Medicine, Seoul National University College of Medicine, Seoul, South Korea; 5 Center for Healthy Society and Education, Seoul National University College of Medicine, Seoul, South Korea; 6 Department of Family Medicine, Seoul National University Hospital, Seoul, South Korea; 7 Department of Biomedical Sciences and Family Medicine, Seoul National University Hospital, Seoul National University College of Medicine, 101 Daehak-ro, Jongno-gu, Seoul, South Korea

**Keywords:** Social capital, Social trust, Cardiovascular disease

## Abstract

**Background:**

Although social capital has been shown to be one of the important social determinants of health, the association between social trust and the risk of cardiovascular disease (CVD) is not clear yet. We aimed to investigate the association of social trust with CVD risk using a large Korean population based data.

**Methods:**

The data of this study was derived from the Korean National Health Insurance Service database. Community-level social trust was determined from the Korean Community Health Survey. The study population consisted of 2,156,829 participants. According to social trust index measured in the area of residence during 2011, participants were followed-up from 1 January 2012 to 31 December 2016. Multivariate Cox proportional hazards regression was used to determine the adjusted hazard ratios (aHRs) and 95% confidence intervals (CIs) for CVD risk according to quintiles of social trust.

**Results:**

Compared to participants with the lowest quintile of social trust, those within the highest quintile had lower risk for CVD (aHR 0.91, 95% CI = 0.89 to 0.93), CHD (aHR 0.92, 95% CI = 0.89 to 0.95), and stroke (aHR 0.90, 95% CI = 0.87 to 0.93). The risk-reducing association of high social trust on CVD risk was preserved after additional adjustments for lifestyle behaviors including smoking, alcohol consumption, and physical activity.

**Conclusion:**

Higher social trust was associated with reduced risk of CVD even after considering lifestyle behaviors. Social trust in a community level is an important determinant of CVD and enhancing social trust may lead to reduced risk of CVD.

## Background

Cardiovascular disease (CVD) is the leading cause of death globally and 17.8 million people died of CVD worldwide in 2012 alone [1]. In South Korea, CVD accounts for 1 every 5 deaths in 2014, and the overall prevalence of atherosclerotic CVD in 2015 was 101.11 per 1000 individuals [2, 3]. The individual risk factors of CVD such as health-related behaviors and biological factors are quite well-known. Meanwhile, beyond the individual level, previous studies have also attempted to determine the risk factors of CVD in the social and community levels. Among these community factors, social capital has been raised as one of the important social determinants of health in recent years.

The definition of social capital has been described by several researchers and it is still debatable [4, 5]. Among a couple of main consented thoughts describing social capital, one from Putnam, who promoted the importance of social capital in the public health field, defined social capital as “features of social organization, such as trust, norms, and networks that can improve the efficiency of society by facilitating coordinated actions” [6]. While there are multiple features of social capital, it can be distinguished by structural or cognitive dimensions. Trust is the main component of cognitive dimension which reflects the community level of social capital [7].

Previous studies have investigated the association of social capital with several health outcomes [6, 8–10]. Based on those studies, recently, a local government in Korea has started a project to promote social capital to improve the health outcomes including CVD in the elderly and is waiting for the results [11]. However, studies on the relationship between social capital and CVD are insufficient and the results are inconsistent. According to a systemic review of studies in adulthood in 2014, it was suggested that social capital was not associated with CVD risk [12]. In terms of studies of each component on social capital, there are very few studies on social trust and CVD and one study showed no significant association between social trust and CVD risk in the general population aged from 18 to 80 [13]. Meanwhile, there are some studies that high social participation seems to be related with low CVD risk in adults aged over 18 years [5, 13–15]. Moreover, most previous studies had relatively small study population sizes and were carried out only in European countries.

Therefore, we aimed to investigate the association of social trust with CVD risk among a large Asian population in South Korea.

## Methods

### Study population

The study population was derived from the Korean National Health Insurance Service (NHIS) database. In South Korea, the NHIS provides mandatory health insurance for all Korean citizens providing nearly all types of health services [16]. Furthermore, the NHIS provides health screening examinations for all enrollees aged 40 years or older, which include a self-reported questionnaire on health-related behaviors, anthropometric measurements, and laboratory exams for blood and urine. The NHIS collects health service utilization information on all insured health services including outpatient and inpatient hospitalizations, health screening examinations, diagnostic and treatment-related procedures, and pharmaceutical prescriptions for claims purposes [17]. From this database, the NHIS provides a part of their data for research purposes. A number of previous large-scale epidemiological studies using NHIS database has been reported and its validity is described in detail elsewhere [17, 18].

Among 2,338,093 participants without previous CVD in 7 metropolitan areas in South Korea aged 59 years or older in 2011, we excluded 31,540 participants who died before the index date of 1 January 2012. Then, 1 participant who did not have the value of social trust and 149,723 who had missing values for covariates were excluded. The final study population consisted of 2,156,829 participants. Starting from 1 January 2012, all participants were followed-up for CVD or death until 31 December 2016.

### Key variables

Social trust values were derived from the Korean Community Health Survey (KCHS) [19], which is conducted by the Korean Centers for Disease Control and Prevention in 2011. KCHS is a nationally-representative community-based cross-sectional survey that contains community-level information according to administrative district sites. Among the survey questions, all participants were asked “Do you trust members of your community?” as a measure of social trust [20]. The proportion of those who answered ‘yes’ to the social trust question for each administrative district site was calculated and determined as social trust. A total of 253 district sties, with a mean (standard deviation) land area of 55.1 (79.9) km^2^, covers the entire South Korea land mass [21]. Social trust in 2011 were then merged according to each participants’ area of residence, which is also categorized into the same administrative district site. The study population was then divided into quintiles according to social trust values, with the first quintile containing participants residing in areas with the lowest social trust values.

Upon admission, the attending physician is required to insert the International Classification of Diseases, Tenth Revision (ICD-10) codes for the primary disease in which the patient was hospitalized for. CVD was defined when a participant was hospitalized for ICD-10 codes for coronary heart disease (CHD) or stroke for 2 or more days. The ICD-10 codes for CHD and stroke were I20 to I25 and I60 to I69, respectively. Multiple previous studies that used the NHIS database had a similar operational definition for CVD [18]. The ICD-10 codes for CVD, CHD, and stroke were adopted from the American Heart Association guidelines [22].

Upon multivariate Cox proportional hazards regression, we adjusted for potential confounding factors including age (continuous, years), sex (categorical, men or women), area of residence (categorical, capital or metropolitan city), household income (categorical, 1st, 2nd, 3rd, and 4th quartiles), and Charlson comorbidity index (continuous). Household income quartile was determined from the insurance premium. Among participants who underwent health examinations within 2 years prior to the index date, we additionally adjusted for lifestyle behaviors and CVD risk factors including smoking (categorical, never, former, and current smokers), alcohol intake (categorical, 0, 0–1, 1–2, 3–4, and 5 times per week), physical activity (categorical, 0, 1–2, 3–4, 5–6, and 7 times per week), body mass index (continuous, kg/m^2^), systolic blood pressure (continuous, mmHg), fasting serum glucose (continuous, mg/dL), and total cholesterol (continuous, mg/dL). Body mass index was calculated by dividing the weight in kilograms by height in meters squared.

### Statistical analysis

The Chi-squared test for categorical variables and analysis of variance for continuous variables were used to determine the difference in characteristics of the study population according to quintiles of social trust. Multivariate Cox proportional hazards regression was used to calculate the adjusted hazard ratios (aHRs) and 95% confidence intervals (CIs) for CVD risk according to quintiles of social trust. Stratified analysis for the association of social trust with CVD risk according to age, sex, household income, and Charlson comorbidity index was conducted. The risk for CVD according to social trust upon additional adjustment for lifestyle behaviors and CVD risk factors among those who underwent health examinations was determined. Finally, subgroup analysis on the association of social trust with CVD risk using those population who did health screening test, was conducted by stratified analysis according to level of smoking, alcohol consumption, and physical activity was also conducted.

Statistical significance was determined as a *p* value of < 0.05 in a two-sided manner. All data collection and statistical analysis were conducted using SAS 9.4 (SAS Institute, Cary, NC, USA).

## Results

Table 1 depicts the descriptive characteristics of the study population. The range for social trust for the first, second, third, fourth, and fifth quintiles were 41.5–52.8%, 53.1–58.3%, 58.5–60.2%, 60.4–66.6%, and 67.6–88.5%, respectively. Compared to participant within the lowest quintile of social trust, those within the highest quintile had higher proportions of those residing in metropolitan cities than those in the capital city, higher household income, and more comorbidities (all *p* values < 0.001).

**Table 1:** Descriptive characteristics of the study population

	Social trust, quintiles					***p*** value
	1st (lowest)	2nd	3rd	4th	5th (highest)	
Social trust range, %	41.5–52.8	53.1–58.3	58.5–60.2	60.4–66.6	67.6–88.5	
Number of people	434,166	444,249	417,097	429,368	431,949	
Age, years, mean (SD)	67.3 (7.1)	67.1 (7.1)	67.4 (7.2)	67.4 (7.1)	67.4 (7.3)	< 0.001
Sex, N (%)						
Men	201,353 (46.4)	207,478 (46.7)	190,434 (45.7)	197,940 (46.1)	199,701 (46.2)	< 0.001
Women	232,813 (53.6)	236,771 (53.3)	226,663 (54.3)	231,428 (53.9)	232,248 (53.8)	
Area of residence, N (%)						
Capital	286,828 (66.1)	212,645 (47.9)	258,848 (62.1)	171,474 (39.9)	58,866 (13.6)	< 0.001
Metropolitan city	147,338 (33.9)	231,604 (52.1)	158,249 (37.9)	257,894 (60.1)	373,083 (86.4)	
Household income, quartiles, N (%)						
1st (highest)	151,583 (34.9)	164,470 (37.0)	167,764 (40.2)	160,742 (37.4)	176,961 (41.0)	< 0.001
2nd	105,931 (24.4)	109,409 (24.6)	97,325 (23.3)	106,419 (24.8)	106,706 (24.7)	
3rd	79,459 (18.3)	78,192 (17.6)	67,796 (16.3)	73,518 (17.1)	69,518 (16.1)	
4th (lowest)	97,193 (22.4)	92,178 (20.8)	84,212 (20.2)	88,689 (20.7)	78,764 (18.2)	
Charlson comorbidity index, N (%)						
0	190,708 (43.9)	193,912 (43.7)	184,037 (44.1)	183,936 (42.8)	180,981 (41.9)	< 0.001
1	121,047 (27.9)	123,748 (27.9)	114,967 (27.6)	118,112 (27.5)	122,152 (28.3)	
≥ 2	122,411 (28.2)	126,589 (28.5)	118,093 (28.3)	127,320 (29.7)	128,816 (29.8)	

*p* value calculated by Chi-squared test for categorical variables and analysis of variance for continuous variables

Acronyms: *SD* standard deviation, *N* number of people

The association of social trust with CVD is shown in Table 2. Compared to those within the lowest quintile of social trust, residents within the highest quintile had lower risk for CVD (aHR 0.91, 95% CI 0.89–0.93), CHD (aHR 0.92, 95% CI 0.89–0.95), and stroke (aHR 0.90, 95% CI 0.87–0.93). There was a risk-reducing trend with higher levels of social trust for CVD (*p* for trend < 0.001), CHD (*p* for trend 0.002), and stroke (*p* for trend < 0.001). Table 3 depicts the stratified analysis on the association of social trust with CVD according to age, sex, household income, and Charlson comorbidity index. Consistent associations were observed in every different stratum. Participants within the highest quintile of social trust had lower risk for CVD among those with age < 65 years (aHR 0.91, 95% CI 0.87–0.94) and ≥ 65 years (aHR 0.92, 95% CI 0.90–0.95). Similarly, both men (aHR 0.90, 95% CI 0.88–0.93) and women (aHR 0.91, 95% CI 0.88–0.94) within the highest social trust quintile had lower risk for CVD compared to those within the lowest social trust quintile groups. The risk reducing association for those with higher social trust on CVD was preserved among subgroups divided according to household income and Charlson comorbidity index.

**Table 2:** Hazard ratios for cardiovascular disease according to social trust

	Social trust, quintiles					***p*** for trend
	1st (lowest)	2nd	3rd	4th	5th (highest)	
Cardiovascular disease						
Events	24,000	24,644	23,224	25,349	25,422	
Person-years	2,040,433	2,087,191	1,960,079	2,011,667	2,021,539	
aHR (95% CI)	1.00 (reference)	0.93 (0.91–0.95)	0.96 (0.94–0.98)	0.95 (0.93–0.97)	0.91 (0.89–0.93)	< 0.001
Coronary heart disease						
Events	9551	10,108	8791	10,188	9690	
Person-years	2,040,433	2,087,191	1,960,079	2,011,667	2,021,539	
aHR (95% CI)	1.00 (reference)	0.93 (0.90–0.96)	0.94 (0.91–0.97)	0.95 (0.92–0.98)	0.92 (0.89–0.95)	0.002
Stroke						
Events	12,078	12,096	11,857	12,616	13,068	
Person-years	2,040,433	2,087,191	1,960,079	2,011,667	2,021,539	
aHR (95% CI)	1.00 (reference)	0.93 (0.90–0.95)	0.97 (0.95–0.99)	0.95 (0.92–0.97)	0.90 (0.87–0.93)	< 0.001

Adjusted hazard ratios calculated by Cox proportional hazards regression after adjustments for age, sex, area of residence, household income, and Charlson comorbidity index

Acronyms: *aHR* adjusted hazard ratio, *CI* confidence interval

**Table 3:** Stratified analysis on the association of social trust with cardiovascular disease according to age, sex, household income, Charlson comorbidity index

	Adjusted hazard ratio (95% confidence interval)					***p*** for trend
	Social trust, quintiles					
	1st (lowest)	2nd	3rd	4th	5th (highest)	
Age, years						
< 65	1.00 (reference)	0.93 (0.90–0.97)	0.94 (0.90–0.97)	0.96 (0.93–1.00)	0.91 (0.87–0.94)	0.001
≥ 65	1.00 (reference)	0.94 (0.91–0.96)	0.98 (0.96–1.01)	0.95 (0.93–0.97)	0.92 (0.90–0.95)	< 0.001
Sex						
Men	1.00 (reference)	0.94 (0.92–0.97)	0.97 (0.95–1.00)	0.95 (0.93–0.98)	0.90 (0.88–0.93)	< 0.001
Women	1.00 (reference)	0.92 (0.89–0.94)	0.95 (0.92–0.97)	0.94 (0.91–0.97)	0.91 (0.88–0.94)	< 0.001
Household income						
Upper half	1.00 (reference)	0.93 (0.90–0.95)	0.96 (0.93–0.98)	0.95 (0.92–0.97)	0.90 (0.87–0.92)	< 0.001
Lower half	1.00 (reference)	0.94 (0.91–0.97)	0.96 (0.93–0.99)	0.94 (0.91–0.97)	0.92 (0.91–0.97)	< 0.001
Charlson comorbidity index						
0	1.00 (reference)	0.95 (0.90–0.99)	0.94 (0.89–0.99)	0.94 (0.89–0.99)	0.89 (0.84–0.94)	< 0.001
1	1.00 (reference)	0.92 (0.89–0.96)	0.95 (0.92–0.99)	0.92 (0.88–0.96)	0.89 (0.85–0.93)	< 0.001
≥ 2	1.00 (reference)	0.93 (0.91–0.95)	0.97 (0.94–0.99)	0.96 (0.93–0.98)	0.92 (0.89–0.94)	< 0.001

Adjusted hazard ratios calculated by Cox proportional hazards regression after adjustments for age, sex, area of residence, household income, and Charlson comorbidity index

Table 4 shows the risk for CVD according to social trust among those who underwent health examinations. After additional adjustment for lifestyle behaviors and CVD risk factors, participants within the highest quintile of social trust had lower risk for CVD (aHR 0.90, 95% CI 0.88–0.93), CHD (aHR 0.91, 95% CI 0.87–0.95), and stroke (aHR 0.90, 95% CI 0.86–0.94) compared to those with the lowest quintile of social trust. Finally, the stratified results using subgroup analysis, in which healthy behavior information existed, for the association of social trust with CVD according to different level per each healthy lifestyle are shown in Table 5. Never smokers had lower CVD risk (aHR 0.89, 95% CI 0.86–0.93) than past or current smokers (aHR 0.91, 95% CI 0.87–0.96) within the highest quintile of social trust. Similarly, all subgroups according to consumption of alcohol or physical activity had lower risk for CVD among those within the highest quintile of social trust compared to those within the lowest quintile of social trust.

**Table 4:** Hazard ratios for cardiovascular disease according to social trust among participants who underwent health examinations

	Social trust, quintiles					***p*** for trend
	1st (lowest)	2nd	3rd	4th	5th (highest)	
Cardiovascular disease						
Events	12,590	13,784	12,634	14,339	14,293	
Person-years	1,210,307	1,288,009	1,180,296	1,247,028	1,274,142	
aHR (95% CI)	1.00 (reference)	0.93 (0.91–0.96)	0.97 (0.94–0.99)	0.94 (0.91–0.97)	0.90 (0.88–0.93)	< 0.001
Coronary heart disease						
Events	5481	6184	5170	6269	5876	
Person-years	1,210,307	1,288,009	1,180,296	1,247,028	1,274,142	
aHR (95% CI)	1.00 (reference)	0.93 (0.89–0.97)	0.93 (0.89–0.97)	0.94 (0.90–0.98)	0.91 (0.87–0.95)	0.003
Stroke						
Events	5968	6365	6125	6758	7060	
Person-years	1,210,307	1,288,009	1,180,296	1,247,028	1,274,142	
aHR (95% CI)	1.00 (reference)	0.93 (0.89–0.97)	0.98 (0.94–1.02)	0.94 (0.90–0.98)	0.90 (0.86–0.94)	< 0.001

Adjusted hazard ratios calculated by Cox proportional hazards regression after adjustments for age, sex, area of residence, household income, Charlson comorbidity index, smoking, alcohol consumption, physical activity, body mass index, systolic blood pressure, fasting serum glucose, and total cholesterol

Acronyms: *aHR* adjusted hazard ratio, *CI* confidence interval

**Table 5:** Stratified analysis on the association of social trust with cardiovascular disease according to lifestyle behaviors among those who underwent health examinations

	Adjusted hazard ratio (95% confidence interval)					***p*** for trend
	Social trust, quintiles					
	1st (lowest)	2nd	3rd	4th	5th (highest)	
Smoking						
Never smoker	1.00 (reference)	0.92 (0.89–0.95)	0.95 (0.92–0.98)	0.93 (0.89–0.96)	0.89 (0.86–0.93)	< 0.001
Past or current smoker	1.00 (reference)	0.95 (0.91–0.99)	0.99 (0.95–1.03)	0.96 (0.92–1.01)	0.91 (0.87–0.96)	0.004
Alcohol intake						
No	1.00 (reference)	0.92 (0.89–0.96)	0.96 (0.93–0.99)	0.93 (0.90–0.97)	0.90 (0.87–0.94)	< 0.001
Yes	1.00 (reference)	0.95 (0.91–0.99)	0.97 (0.93–1.02)	0.96 (0.91–1.00)	0.90 (0.86–0.95)	0.002
Physical activity						
No	1.00 (reference)	0.93 (0.90–0.96)	0.97 (0.93–1.00)	0.92 (0.89–0.96)	0.90 (0.87–0.94)	< 0.001
Yes	1.00 (reference)	0.94 (0.90–0.99)	0.97 (0.92–1.01)	0.97 (0.93–1.01)	0.90 (0.86–0.95)	0.003

Adjusted hazard ratios calculated by Cox proportional hazards regression after adjustments for age, sex, area of residence, household income, Charlson comorbidity index, smoking, alcohol consumption, physical activity, body mass index, systolic blood pressure, fasting serum glucose, and total cholesterol

## Discussion

In this large, population-based retrospective cohort study, higher social trust was associated with lower risk for cardiovascular events. All CHD, stroke and CVD risk were reduced in groups with higher social trust and the results were significant even after multivariate adjustments including lifestyle behavior such as smoking, alcohol intake, and physical activity. To our knowledge, this is the first nationwide cohort study to demonstrate an association between social trust and CVD risk for a large general population of more than 2 million participants.

According to a recent study, social trust was associated with metabolic syndrome [23]. It suggests that low social trust may affect high incidence of metabolic syndrome, and metabolic syndrome increase the risk of CVD. The mechanism of the association of social trust with health have been proposed usually by three casual pathways [24]. First, by increasing social stress and anxiety, low social trust may stimulate the hypothalamic-pituitary-adrenal axis, resulting in high levels of blood cortisol, of which chronic exposure could be a risk factor for CVD [13, 25]. In this psychosocial pathway, high social trust may contribute to reduce CVD risk by decreasing social stress and/or anxiety. Second, high social trust could promote the exchange of health information and access to resources outside the individual’s own network [26]. Third, by encouraging healthy norms and attitudes regarding health-related behavior, high social trust influences adherence to long term healthy lifestyle including physical and nutrition and enhance self-management [24, 26, 27]. Considering possible confounding effects of health behavior, we adjusted lifestyle in a subgroup who underwent health examination and stratified the population in accordance with health behavior including smoking, alcohol intake, and physical activity, and adjusted other behaviors as well. However, in the both subgroup and stratified analysis, the CVD risk-reducing effect of high social trust was preserved and even more significantly among those with healthy behaviors. This study result may bring our attention to the psychosocial pathway, health service/information utilization pathway and/or other possible unknown mechanisms rather than healthy lifestyle pathways.

Other studies on the effect of social trust on CVD risk showed different results from our study. A longitudinal study on the association between social trust and acute myocardial infarction showed a lack of a significant association [13] and a systematic review in 2014 also concluded no association between social trust with CVD risk [12]. More recently, a cohort study in Sweden reported that the negative correlation between social trust and CVD mortality was no longer significant after adjustment for lifestyles and BMI [5]. Although another study in Finland showed that high interpersonal trust was related with CVD mortality, it was significant only among women (HR 0.69, 95% CI 0.51–0.93) and the trust measured at an interpersonal level [28]. The differing results from those from our study may be affected by the study designs of them. While other studies measured social trust by questionnaire and enrolled only who responded to it, our study population was derived from a national wide cohort database and social capital was measured in a district representative sample and transferred to the area level value for all population. Because the response rate of questionnaire may be higher in the population who are healthier or more favorable to the survey compared to the general population, our study from a large population using an area level value from district representative sample for social trust may be less biased in selection and more representative than previous studies. Therefore, our study presents strong evidence that social trust in a community as a dimension of social capital has a CVD risk-reducing effect for the first time.

There were some other studies on social participation, which is another dimension of social capital, and CHD or stroke, in which high social participation was associated with lower risk for CVD [14, 15, 28, 29]. Sundquists et al. used the voting rate of communities as an indirect measure of social capital in his studies in 2006 and 2014 [14, 15]. The results showed that higher social capital was associated with low incidence of CHD and stroke for 9 years follow up [14] and low risk of CHD for 2 years follow up [15]. A similarly designed study using election participation rate and registered crime rate as index of social capital also show the risk-reducing effect of high social capital [29]. Although these two studies were focusing on different dimension of social capital (social participation) while this study focused on another dimension of social capital (social trust), they showed that high social capital (measured by social participation) seemed to be related with low risk of CVD. Also, an individual level cohort study in 2019 and 2007 presented that self-reported high social participation was associated with lower risk for CVD mortality [5, 28].

Our result on the association between social trust and CVD risk suggests that social capital is an important determinant for CVD and policy efforts enhancing social capital to reduce CVD risk is also important. According to a study from Iran, social capital can be improved in communities by planning to improve education and occupation status, paying more attention to strengthening family bonds, and provision of local facilities [30]. Therefore, there is a need for further intervention studies on what intervention can increase social capital and whether attempts at enhancing social capital in a community level is effective on reducing a risk of CVD or not.

The main strength of our study is that in contrast to previous studies on social trust and CVD, selection bias was minimized. Using an area level value for social capital, only a minor proportion of the study population were excluded. Second, as the study population was limited to 7 metropolitan areas in South Korea, we could exclude the area level confounding factors such as urbanization or green space which are quite different between urban and rural by including only urban population [21]. A large population-based urban population representative data may generalize our results than previous non-representative data based research results. Third, we could assess the information for evaluating CVD outcomes such as lifestyle behavior, BMI and underlying disease. Thus, it was possible to adjust for confounding effects CVD events.

There are several limitations in this study. First, social capital was measured once at a point and possible change in trust level was not considered in this study. However, the changes in trust are thought to be ignorable because the communities in a country shared the same social events which could be effective on change of social trust and it has been shown that the values of trust tend to revert back to the initial levels [31]. Second, the social trust was measured in a community level and the participants in the same district have the same level of social trust. Therefore, stratified analysis by region was not able in this study. It is necessary to analyze with the individual-level social trust in future studies. Third, the large population database does not include some demographic factors such as education, marital status and employment status which may effect on CVD risk. Fourth, we excluded missing data instead of other methods such as imputation. It may lead to reduced statistical efficiency of estimates while increasing the potential for bias. However, because the excluded data accounted for only less than 6.5% of all, it is expected that bias is not high. Finally, although this was a large urban representative cohort study, there are still possibility of endogeneity problem and cannot infer a causality of social trust on CVD. However, unless the study is a randomized controlled design, causal inference can be insufficient.

## Conclusions

Higher social trust was associated with reduced risk of CVD. The association was preserved even after adjustments for CVD risk factors such as lifestyle behavior. This suggests that the social capital especially trust in a community is an important determinant of CVD. Therefore, health care policy aimed at enhancing social capital may be beneficial in reducing CVD risk.

## Data Availability

The data that support the findings of this study are available from the Korean NHIS but restrictions apply to the availability of these data, which were used under license for the current study, and so are not publicly available. Data are however available from the authors upon reasonable request and with permission of the Korean NHIS.

## Abbreviations

aHRs: adjusted hazard ratios
CHD: Coronary heart disease
CIs: Adjusted hazard ratios
CVD: Cardiovascular disease
ICD-10: International Classification of Diseases, Tenth Revision
KCHS: Korean Community Health Survey
NHIS: National Health Insurance Service
